# 

**Published:** 1906-06-09

**Authors:** 


### MEDICAL APPOINTMENTS AND VACANCIES.

### APPOINTMENTS.

ASHTON, GEORGE, M.D., Manchester, M.R.C.S. Eng., L.R.C.P. Lond., has been reappointed medical officer for home patients to the Manchester Royal Infirmary.

The following house appointments have been made at St. Thomas's Hospital:—

*Casualty Officers* (from July 2nd, 1906).—(Senior) F. R. E. Wright, M.B. Lond., M.R.C.S., L.R.C.P., D.P.H.; (Junior) L. E. C. Norbury, M.B., B.S., M.R.C.S., L.R.C.P.

*Resident House Physicians.*—F. M. Bulley, M.R.C.S., L.R.C.P., M.A. Cantab (extension); R. C. Jewesbury, M.A., M.B., B.Ch., Oxon., M.R.C.S., L.R.C.P. (extension); M. A. Cassidy, M.A., M.B., B.C. Cantab, M.R.C.S., L.R.C.P.; H. S. Sington, M.R.C.S., L.R.C.P.

*House Physicians to Out-Patients.*—H. C. Squires, M.A., M.B., B.Ch. Oxon., M.R.C.S., L.R.C.P.; A. N. Dickson, M.R.C.S., L.R.C.P.; N. R. Cunningham, M.R.C.S., L.R.C.P.; W. O. Sankey, M.R.C.S., L.R.C.P.

*Resident House Surgeons.*—H. T. Gray, B.A., B.C. Cantab, M.R.C.S., L.R.C.P. (extension); A. W. Hooker, M.B., B.S. Lond., M.R.C.S., L.R.C.P. (extension); J. H. Drew, M.B., B.S. Lond., M.R.C.S., L.R.C.P. (extension); H. Falk, B.A., M.B., B.C. Cantab., M.R.C.S., L.R.C.P. (extension).

*House Surgeons to Out-Patients.*—R. J. H. Cox, M.R.C.S., L.R.C.P. (extension); F. S. Hewett, B.A. Cantab., M.R.C.S., L.R.C.P. (extension); A. B. Howitt, B.A. Cantab., M.R.C.S., L.R.C.P. (extension); W. G. Howarth, M.A., M.B., B.C. Cantab., M.R.C.S., L.R.C.P. (extension).

*Obstetric House Physicians.*—(Senior) S. R. Gibbs, M.R.C.S., L.R.C.P.; (Junior) E. L. Atkinson, M.R.C.S., L.R.C.P.

*Ophthalmic House Surgeons.*—(Senior) C. R. B. Eyre, M.R.C.S., L.R.C.P.; (Junior) H. E. Gotelee, M.R.C.S., L.R.C.P.

*Special Departments.*—(Throat) S. G. MacDonald, B.A. Cantab., M.R.C.S., L.R.C.P. (extension), and H. B. Whitehouse, M.R.C.S., L.R.C.P. (extension); (Skin) J. Wallace, B.A. Oxon., M.R.C.S., L.R.C.P., and G. L. Webb, B.A., B.C. Cantab., M.R.C.S., L.R.C.P.; (Ear) A. L. Loughborough, M.R.C.S., L.R.C.P. (extension), and G. D. Alexander, B.A. Cantab., M.R.C.S., L.R.C.P.

*Children's Surgical.*—C. M. Page, M.R.C.S., L.R.C.P. (extension); S. G. Macdonald, B.A. Cantab., M.R.C.S., L.R.C.P. (extension).

### VACANCIES.

BRECKNOCK COUNTY AND BOROUGH INFIRMARY.—Resident House Surgeon.

BROMSGROVE, WORCESTERSHIRE, BARNSLEY HALL ASYLUM.—Medical Superintendent.

CANCER HOSPITAL, Fulham Road, London, S.W.—Surgical Registrar.

DEVONPORT, ROYAL ALBERT HOSPITAL.—Assistant Resident Medical Officer.

EAST LONDON HOSPITAL FOR CHILDREN AND DISPENSARY FOR WOMEN, Shadwell, E.—Surgeon. Also Assistant Surgeon.

GORODN HOSPITAL FOR FISTULA, ETC., Vauxhall Bridge Road, S.W.—Honorary Anæsthetist.

HANTS COUNTY ASYLUM.—Third Assistant Medical Officer.

INDIAN MEDICAL SERVICE.—Examination for not less than Twenty Commissions.

LITTLEMORE PAUPER LUNATIC ASYLUM, near Oxford.—Second Assistant Medical Officer.

LIVERPOOL STANLEY HOSPITAL.—Assistant Honorary Ophthalmic Surgeon.

MIDDLESBROUGH, NORTH RIDING INFIRMARY.—Assistant House Surgeon.

NORFOLK AND NORWICH HOSPITAL.—Second Assistant House Surgeon.

NORTHAMPTON, SAINT ANDREW'S HOSPITAL FOR MENTAL DISEASES.—Junior Assistant Medical Officer.

ROYAL FREE HOSPITAL, Gray's Inn Road, W.C.—Anæsthetist. Also Assistant Anæsthetist and Clinical Assistants.

SALFORD ROYAL HOSPITAL. Junior House Surgeon.

STOCKPORT INFIRMARY.—Junior Assistant House Surgeon.

STROUD GENERAL HOSPITAL.—House Surgeon.

WEST AFRICA, PRINCESS CHRISTIAN HOSPITAL, Sierra Leone.—Medical Officer.

WEST-END HOSPITAL FOR DISEASES OF THE NERVOUS SYSTEM, 73 Welbeck Street, W.—Resident House Physician and Medical Registrar.

WOLVERHAMPTON AND MIDLAND COUNTIES EYE INFIRMARY.—House Surgeon.

### THE INTERNATIONAL LIBRARY.

Our readers who have literary tastes especially should avail themselves of the offer of *Lloyd's News* to send gratis and post free a descriptive book of 124 pages to anyone who chooses to apply for it. This free book tells the story of the International Library of 20 large volumes, and contains many illustrations. The demand for the book has been enormous, and a final edition of it has just been issued. Further particulars appear on page iii.

### The Care of Children

(See Announcement on page xi.)

The following new Book will be found of the greatest value to those Nurses who are taking up this important branch of the Nursing Profession :—

### THE CARE OF CHILDREN:

**Practical Hints for Mothers and Nurses at Home and Abroad.**

BY

**ROBT. J. BLACKHAM, D.Ph. (Lond.).**

**Price 1/6 net, 1/8 post free.**

Contains:—Hints on Food, Drink, and Medicines, Clothing, Cleanliness, Rest and Exercise, Infectious Diseases, on the Nursery, on Nursery "First Aid," on Nursery Cooking, &c. &c.

### THE MOTHER'S HELP AND GUIDE TO THE DOMESTIC MANAGEMENT OF HER CHILDREN.

By P. Murray Braidwood, M.D. 2/- post free.

### NURSING OF SICK CHILDREN.

By D. Burnet. 1/- net, 1/1 post free.

### SIGHT AND HEARING IN CHILDHOOD.

By R. Brudenell Carter, F.R.C.S., and Arthur H. Cheatle, F.R.C.S. 2/- net, 2/3 post free.

### SPINAL CURVATURE AND AWKWARD DEPORTMENT: THEIR CAUSES AND PREVENTION IN CHILDREN.

By Dr. George Müller, Professor of Medicine and Orthopædics, Berlin. English Edition, edited and adapted by Richard Greene, F.R.C.P. Ed.
With Plates and Illustrations, 2/6 post free.

### SOME HEALTH ASPECTS OF EDUCATION.

By Percy G. Lewis, M.D., M.R.C.S,, L.S.A., Author of "Nursing: Its Theory and Practice." Crown 8vo. paper cover, 1/- post free.

### CLEANLINESS IN CHILDREN.

By Rose Petty, Associate of the Sanitary Institute.
Crown 8vo. paper cover, **2d.** post free.

**The Scientific Press, Ltd.,**

*Catalogue of Nursing Manuals sent post free on application.*

**Publishers of Nursing Text-Books, Charts, and Case-Books of all Descriptions.**

**28 & 29 SOUTHAMPTON STREET, STRAND, LONDON, W.C.**

### Publications.

### NEURASTHENIA, Clinical Lectures on.

**By THOMAS D. SAVILL, M.D. Lond.** 2nd Edition.

*Physician to the Hospital for Diseases of the Nervous System, &c. &c.*

"Cannot fail to be a useful guide to the diagnosis and treatment of a prevalent disease."—The Hospital. "Evidently the result of long study and observation."—Med. Journ. California.

H. J. GLAISHER, 57 Wigmore Street, W. **5s. net; 5s. 4d. by post.**

### DISEASES OF THE ANUS & RECTUM

**By D. H. GOODSALL, F.R.C.S., and W. ERNEST MILES, F.R.C.S.,**

Senior Surgeon Metropolitan Hospital; late Senior Surgeon, St. Mark's Hospital. Surgeon Cancer Hospital; Surgeon Gordon Hospital.

PART I.—Containing Chapters on Anatomy, General Diagnosis, Abscess, Ano-Rectal Fistula, Recto-Urethral, Recto-Vaginal and Recto-Vesical Fistula, Sinus over the Sacrum and Coccyx, Anal Fissure and Hæmorrhoids (or Piles). With 91 Illustrations, 8vo. 7s. 6d. net.

PART II.—Containing Chapters on Prolapse, Invagination Ulceration, Stricture, Malignant Growths, Benign Tumours, Foreign Bodies, Pruritis Ani, and Syphilis. With 44 Illustrations, 8vo. 6s. net.

**LONGMANS, GREEN & CO., London, New York, Bombay.**

Recently Published, Demy 8vo. cloth, 7s. 6d.

### THE INFLAMMATION IDEA IN GENERAL PATHOLOGY.

**By W. H. RANSOM, M.D., F.R.S., &c.**

"The Essay has more than weight enough to recommend itself to the consideration of skilled inquirers specially interested in its problems, for it bears on every page evidences of original observation and independent thought."—*Scotsman.*

WILLIAMS & NORGATE, 14 Henrietta St., Covent Garden London, W.C.

### WORKS BY DAVID WALSH, M.D.

**RÖNTGEN RAYS IN MEDICAL WORK.** *Third Edition.* 12s. 6d.

**THE HAIR AND ITS DISEASES.** 2s. 6d. net. A Standard Monograph. Baillière, Tindall & Cox, London.

**GOLDEN RULES OF SKIN PRACTICE.** 1s. net. *Second Edition.* Wright & Co., Bristol.

**AGE AND OLD AGE: Handbook.** 2s. 6d. R. A. Everett & Co. London.

JUST PUBLISHED. Price **2s. 6d.** net.

### CARCINOMA OF THE RECTUM.

**By F. SWINFORD EDWARDS, F.R.C.S.,**

Surgeon to the West London Hospital; Senior Surgeon to St. Peter's Hospital for Stone, and to St. Mark's Hospital for Fistula, &c.

London: Baillière, Tindall, & Cox, 8 Henrietta Street, Covent Garden

THE MOST EFFECTUAL BANDAGE FOR THE CURE OF ULCERS AND OTHER DISEASES OF THE LEG.

### THE POCKET-BOOK OF TEMPERATURE CHARTS.

Containing Seventeen 12-hour and eight 4-hour Charts, perforated so that each Chart may be easily removed after use.

Bound in paper cover. Suitable size for the pocket. Price **6d.** net; by post **7d.**

*Of all Booksellers, or of*
THE SCIENTIFIC PRESS, LIMITED, 28 & 29 Southampton Street, Strand, London, W.C.

